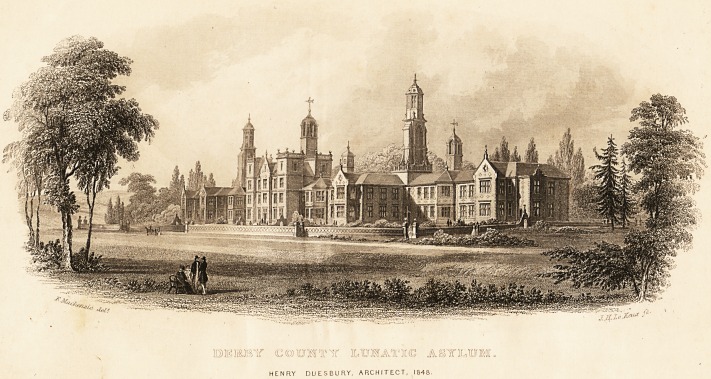# Lunatic Asylums of Ireland

**Published:** 1853-01-01

**Authors:** 


### DERBY COUNTY LUNATIC ASYLUM.

HENRY DUESBURY, ARCHITECT, 1848.

### LUNATIC ASYLUMS OF IRELAND.

In Dr. Fitzgerald's annual report of the Limerick District Lunatic Asylum, embracing the three counties of Limerick, Clare, and Kerry, for the year ended 31st March, 1852, we find the following summary of the year's proceedings contained in "Table No. 1."

| | Males. | Females. | Total. |
|---|---|---|---|
| Number of patients in the asylum the 1st of April, 1851 . . . . . . . . . . . . | 167 | 170 | 337 |
| Number admitted during the year . . . . | 34 | 32 | 66 |
| Total under treatment during the year . . . | 201 | 202 | 403 |
| Discharged cured and relieved . . . . . . . | 19 | 22 | 41 |
| Ditto not cured . . . . . . . . . . . | 4 | 3 | 7 |
| Died . . . . . . . . . . . . . . . | 8 | 6 | 14 |
| | 31 | 31 | 62 |
| Remaining in the asylum 31st March, 1852 . | 170 | 171 | 341 |

According to "Table No. 2," the total expenditure of the year appears to have been £4399 16s. 7d., and the average annual cost of each patient, including all charges, £12 18s. 0¾d. The employment of the patients is contained in "Table No. 3," which shows the total employed amongst the males as 68; of whom, 56 were engaged in farm and garden labour, and 12 in miscellaneous works. Of the females, 74 were employed; their avocations being needlework (26), knitting (27), assisting in laundry (11), and cleaning the house (10); thus making the total, male and female, employed 165, and unemployed 176. "Table No. 4" shows the produce of the farm and garden during the year to have been valued at £220 19s. "Table No. 5" is devoted to a statement of the work and "repairs" executed by the females, which would appear to have been very considerable, and "No. 6" to carpenter's work, but whether done by inmates or not, is not stated. The last table in the list, "No. 7," has reference to the "comparative amounts annually issued from the treasury for the last five years," which shows that a regular decrease of expenditure has been going on during that period, viz., from £15, in round numbers, to £12, and on which subject the report states that, "although an increased number of patients have been received, it is satisfactory to be able to say that it is by several hundred pounds less than the expenditure of the preceding years, as will be seen by the comparative issues from the treasury as set forth, together with the average cost per head per annum of each patient." And, again, "these financial statements I am well aware would fall far short of being satisfactory, if at the same time I could not assure you that the inmates receive every care and comfort that it is possible to bestow upon them." We are well pleased with this deliberate, and as we take it, *bonâ fide* statement of Dr. Fitzgerald, as there is much danger we are afraid, owing to the clipping-down system of the present day in public institutions, and especially in lunatic asylums, of their inmates being reduced down to the lowest state of maintenance, in order to obtain the favour of the authorities, by showing how much economy has been practised, and how much better, accordingly, that asylum is managed, whose expenditure is at a low figure than another at a higher one, which we must protest against as being a most unfair and disingenuous mode of procedure, and yet the one of all others that is sure to be extolled by the public, and its abettors held up as nothing short of benefactors! But we would fain hope better things of men of enlarged and educated minds, the members of an honourable profession who are placed in immediate care of the insane in public asylums, with whom the primary object always should be, and we trust is, to discharge their important trust towards their unfortunately afflicted patients faithfully and *liberally*, and never sacrifice their interests and well-being at the shrine of economy, for the sake of being smiled upon by rate-payers and boards of governors, or visiting justices. We, however, would have been glad had the dietary table of the Limerick Asylum appeared in this report, so as to enable us to form some criterion of the "care and comfort" that is bestowed, as regards the "creature comforts," on its inmates; it may be on a very liberal scale, and as we suppose it is, from Dr. Fitzgerald's so specially guarding his institution against any imputation of its inmates not "receiving every care and comfort that it is possible to bestow upon them," by reason of the vaunted saving of the "several hundred pounds" referred to; but were we to judge it on this point of detail by other asylums in Ireland, we would have to pronounce it very far short of what it ought to be. In point of ordinary statistical information this report of the Limerick Asylum is exceedingly deficient; for instance, we have no particulars given of the causes of the deaths which occurred during the year, nor of those of derangement, or of the ages of the patients admitted and discharged, or who died, neither of the period of time under treatment prior to being discharged as "cured;" all which and sundry others, we submit should be, as a matter of course, contained in the official reports of hospitals for the insane, and which we hope Dr. Fitzgerald will for the future embody in his, and at the same time "enlarge its borders" a slight degree, as a more lilliputian document of the kind, or one so morally mean in externals, coming from a great public institution, our editorial eyes never before beheld; but which perhaps was necessary in helping to carry out so sedulously and perseveringly that system of expenditure, whose motto is "every little makes a muckle," and which, like everything else that is good in the abstract, may be carried out to a most pernicious extreme in practice.

The Carlow District Hospital for the insane poor of the counties of Carlow, Kildare, Wexford, and Kilkenny, is superintended by Dr. W. E. White, from whose nineteenth annual report, for the year ended 31st March, 1852, we collect that there remained in hospital on the 1st April, 1851, 107 males and 90 females; since which, 29 new cases of males and 30 females had been admitted, together with four relapses of males and three of females, making a total under treatment during the year, of 263; of whom, 140 were males and 123 females. Those discharged recovered, were 18 males and 14 females; relieved or removed by friends, six males and five females; unfit or incurable, three males and two females. "Discharged died, 10 males and six females." This method of enumerating deaths amongst the *discharges*, we frequently observe in reports, which is palpably wrong, and should be corrected. The number remaining in hospital 31st March, 1852, was 302; of whom, 103 were males and 199 females, Of the patients admitted during the year, 22 were between 20 and 30 years of age, 15 between 15 and 20, and 30 and 40, respectively, 10 between 40 and 50, 3 between 50 and 60, and one under 15. Amongst the causes we find 22 under the head of hereditary predisposition, 8 from anxiety, caused by the state of the times, 6 from fever, &c. &c. In social condition, 37 of the admissions of the year were single, 19 married, 3 widowers, 1 widow, and 7 unknown, and whom, we may reasonably conclude, were of the single class: thus making it more than double that of the married. In point of education, 24 could read and write, 11 were well-educated, 9 could read only; uneducated 1, and unknown 21, which appears strange not to have been able to discover. Of the cases who were discharged, recovered or otherwise, 30 were of those admitted during the year, 6 from one to two years, 2 from two to three years, 4 from three to five years, and 6 were five years and upwards under treatment. The oldest patients amongst those who died were a male and a female, whose ages were each between 50 and 60 years, the youngest from 20 to 30, being four in all; two males and two females. The period of residence of those who died was as follows:—under one year, 5; from one to two years 2; two to five years, 2; five to seven years, 2; seven to ten years, 2; ten years and upwards, 3. The causes of death are thus given:—cerebral congestion, 1; scrofulous disease of the pancreas, 1; scrofulous tubercles in mesentery, 1; dropsy, 1; dysentery, 2; hæmorrhoids, 1; paralysis des alienés, 2; pneumonia, 1; phthisis pulmonalis, 3; maniacal exhaustion, 2; old age and natural decay, 1.

The general dietary is, breakfast 8 oz. of meal made into one quart of stir-about (or porridge), and one-third of a quart of new milk. Dinner, males, $10\frac{2}{3}$ oz. of bread and 1 pint of mixed milk on four days in the week, and the other three days a quart of good beef soup, with the meat and vegetables cut up in it, instead of the milk, and bread the same. The females get the same fare with the exception of the bread, which for them is reduced to 8 oz. The supper for all is half a pound of bread with one pint of mixed milk. The cost incurred in maintaining the establishment during the year amounted to £3084 16s. 1d.; the items of which were—for provisions, £1216 12s. 7d.; groceries, £44 10s. 6d.; clothing, £208 19s.; bedding, £87 1s. 6d; furniture, £43 7s. 11d.; building and repairs, £261 19s. 8d.; soap, starch, gas, &c. £55 3s. 1d.; coals and turf, £166 7s. 2d.; farm and garden, £10 1s.; taxes and insurance, £5 8s. 9d.; books, stationary, and printing, £20 3s. 11d.; medicine, £15 6s. 9d.; wine, porter, and spirits, £18 2s. 10s.; tobacco and snuff, £22 5s. 7d.; salaries, £515; wages, £245 12s.; premiums to servants, £20 7s. 6d.; incidents, £38 6s. 4d. Dr. White makes some very judicious remarks in this report, which bears evidence of being very carefully and creditably written; no important matter in the conduct of the institution being omitted, and much that is suggestive contained in its pages. We are surprised to find from his report that there are only three Irish acres of land under cultivation in his establishment, exclusive of the gardens, the rest of the ground being occupied with buildings, the consequence of which is, he observes, "that for the great portion of the male patients we have literally no employment, as soon as the few busy spring and autumn days are passed." To remedy this great evil—for a great one it is—Dr. White suggests the prudence of taking "a small farm, say from ten to fifteen acres of land, at some conveniently short distance from the asylum. The working men might be marched there every day, under charge of their attendants, after breakfast, and they need not return till evening, as their dinner could be sent to them. This would give occupation, exercise, variety of scene and circumstance; and I am certain all these would be eminently beneficial towards their recovery."

There is novelty and excellent good feeling in this proposal, but we have strong doubts that the multitude of inconveniences and annoyances of various kinds in carrying it out, would more than counterbalance the anticipated good effects from its adoption. Dr. White will excuse us, we are sure, for not falling in exactly with his well intended and humane views on this point; we most cordially concur, however, with him as to the absolute necessity of additional ground being procured, but we would most strongly recommend him to have it, and those employed on it, as immediately as possible under his own eye, otherwise he will find that his daily troubles and cares will be immeasurably added to, and that ultimately the speculation will have to be surrendered as an utterly impracticable one. The annexed paragraph respecting "criminal lunatics," so called, is interesting:

"The removal, some time since, of the criminal lunatics to the Central Asylum at Dundrum, has been attended with very beneficial effects. This class of patients was exeeedingly troublesome in an institution like ours. Many of them imagined their position—'*government patients*,' as they styled themselves, gave them a different status in the house to that of the others; in short, they were '*personages*' in their own opinion, and very difficult personages to manage.

They were frequently quarrelling with the other inmates, and with the attendants; the criminal act they had committed would by some chance or other transpire, and their companions would perhaps allude to it. Hence would arise complaints, and lying, and tale-bearing. Being for the most part either recovered, or only partially and temporarily insane, their character of 'criminal lunatics' made a stricter and more rigid discipline necessary to prevent their escape than would have been at all required, or *even useful*, for ordinary patients. This is now at an end. The former prison character of our hospitals is removed; and we have now the satisfaction of superintending *curative* institutions instead of *gaols*. For all this, as well as for many other changes calculated to improve the position and status of the resident officers of these institutions, we are indebted to Drs. White and Nugent, the inspectors-general of our department, who have been unceasing in their attentions to the interests and comforts of all under their charge. *Sit laus ubi laus debetur.*"

The Maryborough District Lunatic Asylum has for its resident physician Dr. Thomas C. Burton; his report of which, from 1st April, 1851, to 31st March, 1852, shows that the admissions during the year amounted to 42; readmissions, 4; the discharges cured to 27; the deaths to 23; remaining, 31st March, 1852, 188. Dr. Burton states, with reference to the health of the institution during the year, that it "had not been so good as last year, there having been 23 deaths. This had resulted from severely protracted and intractable cases of dysentery; also the admission of cases in a most debilitated condition, and of an advanced age, and the natural consequences of long-continued chronic diseases. In our experience of public hospitals for the insane at home and abroad, the two causes above stated, of increasing their mortality, are generally prevalent ones; dysenteric attacks of a remarkably uncontrollable type, resisting all ordinary treatment, are their bane. As a class, too, the insane do not bear active treatment; and when the subjects of bodily disease the least acute in character, they very rapidly sink under it, be the appliances used as they may. And, as regards the admission of previously debilitated, in fact, completely prostrated constitutions, such are too common, and a source of much annoyance; being sent, as it were, merely to die and be buried at the public charge.

A change in the dietary in use in the Maryborough Asylum, it would appear from a special reference to it by Dr. Burton, has been suggested by the Commissioners of Lunatic Asylums, and certainly we would say that one on a more liberal and nutritive scale would be a vast improvement, and greatly tend towards promoting the general health of the inmates. We are fully aware, on this head, however, that the medical officers charged with the care of the insane experience much difficulty in persuading boards of the absolute necessity there exists of having the standing dietary of a full and nourishing kind, the disease they are the subjects of being for the most part one of atony and debility, and requiring accordingly every care especially in their dietetic treatment. Here is the dietary as at present existing. Breakfast, one quart of stirabout, made of 8 oz. of oatmeal; one-third quart of new milk. Dinner, for four days, 12 oz. bread for males, 10 oz. for females; one pint buttermilk or skim milk: ditto for three days, one quart soup, made of half-a-pound of beef and bone, or beef-heads; bread the same. Supper, 6 oz. white bread, and one-third quart of new milk. The cost of this diet is estimated at 3¼d. per diem.

The employment of the patients, male and female, appears to be very fully provided for and attended to; a statement is given of the produce of the farm during the past year; the saving to the public, caused by the male patients' labour in tilling it, being £138 3s. 5d. The work executed in tailoring and shoemaking is very considerable, to say nothing of the useful and profitable avocations of the females, as duly tabled in the report. Some of the routine practice of the institution is thus set forth, which speaks for itself. "The patients, male and female, alternately take daily exercise on the land and gardens, which are tastefully planted, and laid out with cheering walks. In wet weather, they exercise in the day-rooms, corridors, and walking-sheds. The patients frequently amuse themselves at ball-playing, dancing, music, reading, fancy-work, &c. In the winter months, lights are provided and fires kept up in the day-rooms and corridors, for the employment and amusement of the patients, till the regulated hour for retiring to bed." The cost of maintenance during the year amounted to £3086 12s. 9d.; the patients supported being 192; making the cost per head, on the gross expenditure, £16 1s. 6d.

Having given the above abstract of the Maryborough District Lunatic Asylum Report, we have only further to add, that it occurs to us it would be a very desirable change for the better were its form modernized, an improvement for which, in common with that of the Limerick Asylum, already referred to, there is much need. And, at the same time, if a little more of professional, and considerably less of merely monetary, details were introduced, its value psychologically would be greatly enhanced.

We must not conclude the foregoing brief notices of the Irish district hospitals and houses for the insane, without referring complimentarily to the able and impartial manner in which the official duties connected with them on the part of the Government Commissioners are performed, by Drs. White and Nugent, both of whom, since their appointment to their high and responsible office, have gained for themselves the most unqualified approbation of all parties, for their able and impartial conduct, and the kindly and courteous spirit by which they have been always actuated in carrying into effect the powers vested in their hands. They have been the means of accomplishing many most salutary changes and improvements in the Irish district asylums, which bear so superior a character; and chief among them must be noted that of medical men of station and experience being placed in their charge, and their position in all respects duly attended to, than which nothing could be more to the credit of those gentlemen, or be more to their praise.

---

## Figures and Tables

**Figure f1:**